# Serotonin Type 3 Receptor Is Potentially Involved in Cellular Stress Induced by Hydrogen Peroxide

**DOI:** 10.3390/life12101645

**Published:** 2022-10-19

**Authors:** Ana Salomé Correia, Isabel Silva, José Carlos Oliveira, Henrique Reguengo, Nuno Vale

**Affiliations:** 1 OncoPharma Research Group, Center for Health Technology and Services Research (CINTESIS), Rua Doutor Plácido da Costa, 4200-450 Porto, Portugal; 2 Institute of Biomedical Sciences Abel Salazar (ICBAS), University of Porto, Rua de Jorge Viterbo Ferreira 228, 4050-313 Porto, Portugal; 3 CINTESIS@RISE, Faculty of Medicine, University of Porto, Alameda Professor Hernâni Monteiro, 4200-319 Porto, Portugal; 4 Clinical Chemistry, Department of Laboratory Pathology, Hospital Center of the University of Porto (CHUP), Largo Professor Abel Salazar, 4099-313 Porto, Portugal; 5 Unit for Multidisciplinary Research in Biomedicine (UMIB), University of Porto, Rua de Jorge Viterbo Ferreira 228, 4050-313 Porto, Portugal; 6 Department of Community Medicine, Health Information and Decision (MEDCIDS), Faculty of Medicine, University of Porto, Rua Doutor Plácido da Costa, 4200-450 Porto, Portugal

**Keywords:** serotonin, 5-HT3 receptor, mirtazapine, scopolamine, lamotrigine, hydrogen peroxide, corticosterone

## Abstract

Depression is a disease with several molecular mechanisms involved, such as problems in the serotonergic pathway. This disease is very complex and prevalent, and thus important to deeply study and aim to overcome high rates of relapse and therapeutic failure. In this study, two cellular lines were used (HT-22 and SH-SY5Y cells) to gain insight about the role of the serotonin type 3 (5-HT3) receptor in cellular stress induced by hydrogen peroxide and/or corticosterone. In research, these compounds are known to mimic the high levels of oxidative stress and dysfunction of the hypothalamus–hypophysis–adrenal axis by the action of glucocorticoids, usually present in depressed individuals. The receptor 5-HT3 is also known to be involved in depression, previously demonstrated in studies that highlight the role of these receptors as promising targets for antidepressant therapy. Indeed, the drugs used in this work (mirtazapine, scopolamine, and lamotrigine) interact with this serotonergic receptor. Thus, by using cell morphology, cell viability (neutral red and MTT), and HPLC assays, this work aimed to understand the role of these drugs in the stress induced by H_2_O_2_/corticosterone to HT-22 and SH-SY5Y cell lines. We concluded that the antagonism of the 5-HT3 receptor by these drugs may be important in the attenuation of H_2_O_2_-induced oxidative stress to the cells, but not in the corticosterone-induced stress.

## 1. Introduction

Depression is a complex and common disease worldwide, characterized by several abnormalities such as lack of energy and anhedonia and affecting the daily lives of individuals. In severe cases, death by suicide is a reality. According to the World Health Organization, depression is the most prevalent health problem in the European Union, affecting around 50 million people. Statistics also reveal that 11% of the population will suffer a depressive episode during their lifetime, and that this is the second leading cause of disability. Particularly, Portugal occupies the 5th position among the countries with the most cases of depression: about 8% of the population suffers from the disease [1]. Deficiency in serotonin (5-HT) and other neurotransmitters such as noradrenaline are one of the factors underlying depression, despite the presence of dysregulation of, for example, immune and endocrine factors [2]. In fact, it is an extremely complex disease and is difficult to study because different molecular mechanisms are involved, varying widely between individuals. Indeed, high rates of relapse are associated with depression, highlighting the importance of more research about this disease [3,4]. Cellular studies enable faster and simpler research associated with depression’s pathophysiology at a molecular/cellular level, avoiding the use of animals in the initial stages of research [5]. Thus, in our study, we used two cellular lines: HT-22 (mouse hippocampal neuronal cell line) and SH-SY5Y cells (human neuroblastoma cell line), commonly reported in the study of molecular aspects involved in depression [6,7,8,9]. The potential role of the 5-HT3 receptor in hydrogen peroxide (H_2_O_2_)/corticosterone-induced cellular stress was explored in this study. Several studies report the use of these two compounds to induce cellular stress [10,11,12,13]. Indeed, increased levels of oxidative stress markers and dysfunction of the hypothalamus–hypophysis–adrenal (HPA) axis by the action of glucocorticoids (such as corticosterone) are widely reported in depression [14,15]. In this study, these responses were mimicked by H_2_O_2_ and corticosterone, respectively, as previously reported. 

5-HT3 receptors are ligand-gated ion channels, contrasting with the other subtypes of 5-HT receptors. They are present in the central and peripheral nervous system, being widely expressed in brain stem nuclei and higher cortical areas, such as the hippocampus [16]. Additionally, these receptors are composed of five subunits that surround a central pore, occurring in both homopentameric (only 5-HT3A subunits) and heteropentameric forms (5-HT3A and 5-HT3B receptor subunits) [17]. These receptors are associated with molecular mechanisms present in depression. Indeed, several studies evidenced the role of these receptors as potential targets for antidepressant drugs [18,19]. For example, the application of a selective antagonist of these receptors (ondansetron) to mice demonstrated significant antidepressant-like effects [20]. Some clinical studies also highlighted the role of 5-HT3 receptor antagonists in the reversion of depressive symptoms in humans [16]. 

Mirtazapine, scopolamine, and lamotrigine interact with this receptor. Mirtazapine is a clinically used antidepressant that increases noradrenergic and serotonergic transmission, being an antagonist of adrenergic α2 and the 5-HT2 and 5-HT3 receptors, previously used in our investigation group as a reverser of H_2_O_2_ stress induction [21]. Regarding scopolamine, it is a drug typically used to treat postoperative nausea and vomiting and motion sickness [22], being a non-specific antagonist of acetylcholine muscarinic receptors [23]. Additionally, this drug was also reported to function as an antagonist of 5-HT3 receptors [24]. The potential of scopolamine to be used in treating depression has also been reported [25]. Indeed, in mice, this drug improved depressive symptoms [26]. In clinical studies, scopolamine also revealed rapid antidepressant effects in both unipolar and bipolar depressive individuals [27]. This drug downregulates glutamate receptor production, leading to a decrease of glutamate transmission and decreased excitotoxicity. In addition, by upregulating acetylcholine production, this drug modulates nicotinic, serotonergic, and dopaminergic systems, mechanisms important in antidepressant response [28]. Reports of the interaction of lamotrigine with the 5-HT3 receptor also indicate that this drug inhibits this receptor. Indeed, this drug inhibits currents activated by this receptor, binding to the open state of the channels and blocking channel activation or accelerating desensitization [29,30]. This drug selectively binds and inhibits voltage-gated sodium channels, characterized by being an anti-seizure/anti-epileptic drug, also used in bipolar I disorder [31]. In persistent depression, lamotrigine was also reported to be possibly effective for the treatment of individuals with persistent depression who were antidepressant resistant [32]. A meta-analysis also concluded that this drug was also better than the placebo in improving unipolar and bipolar depressive symptoms [33].

Hereupon, we aimed to study the potential role of drugs that interact with the 5-HT3 receptor (mirtazapine, scopolamine, and lamotrigine) in the attenuation of H_2_O_2_/corticosterone-induced stress to HT-22 and SH-SY5Y cells, mainly by using cell morphology, cell viability, and HPLC assays. Our main findings reveal that the antagonism of the 5-HT3 receptor by these drugs may be relevant in the attenuation of oxidative stress in the cells (H_2_O_2_-induced), but not corticosterone-induced stress.

## 2. Results

### 2.1. Presence of 5-HT3 Receptor on SH-SY5Y and HT-22 Cells

**Figure 1:**
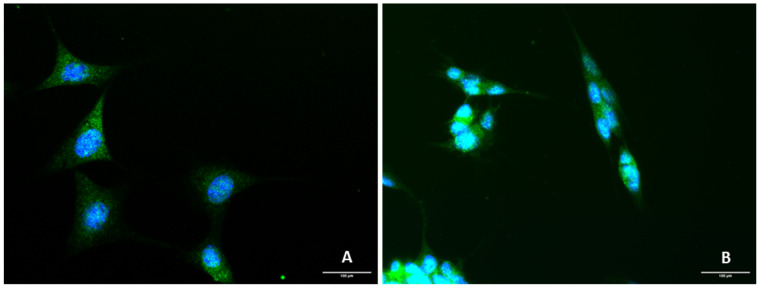
Representative images (400× total magnification) of (**A**) HT-22 and (**B**) SH-SY5Y cells after immunostaining with DAPI (blue) and primary antibody rabbit polyclonal anti-5-HT3AR/donkey anti-rabbit 488 secondary antibody (green). Scale bar: 100 µm.

To demonstrate the presence of the 5-HT3 receptor on both SH-SY5Y and HT-22 cells, immunofluorescence experiments on both cell lines were performed (Figure 1). The procedure is described in the Materials and Methods section. 

Our results demonstrate that 5-HT3A receptor (represented in green) is present in both HT-22 and SH-SY5Y cell lines.

### 2.2. Effect of Mirtazapine, Scopolamine, and Lamotrigine on SH-SY5Y and HT-22 Viability

**Figure 2:**
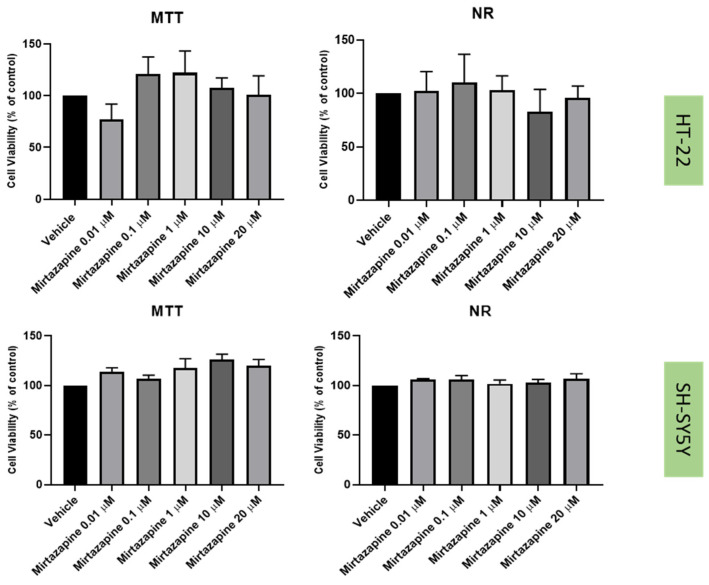
HT-22 and SH-SY5Y viability after incubation of mirtazapine (0.01 µM–20 µM) for 48 h, obtained by MTT (**left**) and NR (**right**) assays. The results represent the mean ± SEM of 3 independent assays. For SH-SY5Y, these results were previously developed by our Research Group [21].

**Figure 3:**
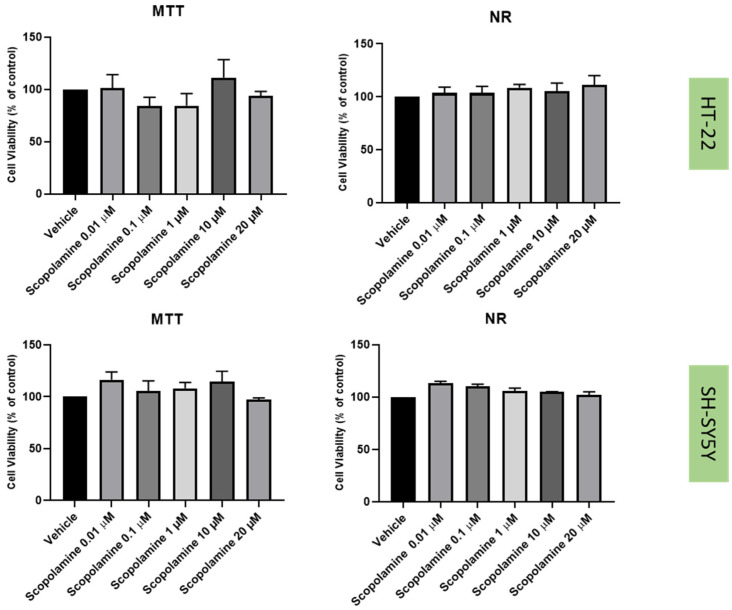
HT-22 and SH-SY5Y viability after incubation of scopolamine (0.01 µM–20 µM) for 48 h, obtained by MTT (**left**) and NR (**right**) assays. The results represent the mean ± SEM of 3 independent assays.

**Figure 4:**
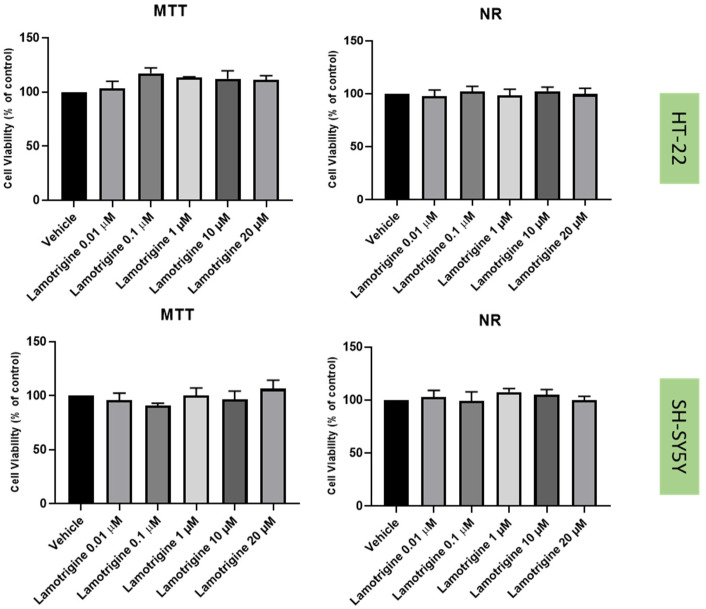
HT-22 and SH-SY5Y viability after incubation of lamotrigine (0.01 µM–20 µM) for 48 h, obtained by MTT (**left**) and NR (**right**) assays. The results represent the mean ± SEM of 3 independent assays.

**Figure 5:**
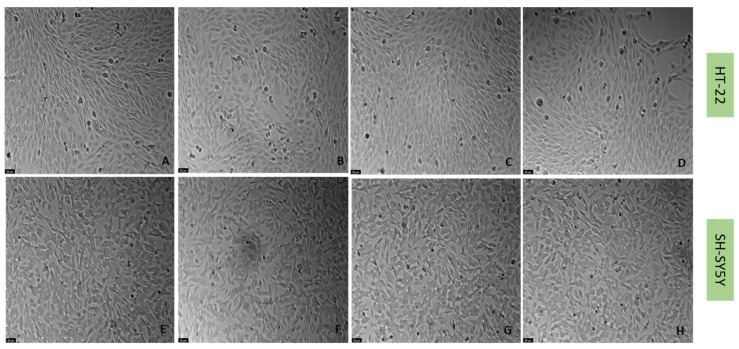
HT-22 and SH-SY5Y morphology after incubation of (**A**,**E**) vehicle, (**B**,**F**) mirtazapine 20 µM, (**C**,**G**) scopolamine 20 µM, and (**D**,**H**) lamotrigine 20 µM, for 48 h (100× total magnification). Scale bar: 50 µm.

To understand the effects on the cellular viability of three drugs that interact with the 5-HT3 receptor, mirtazapine (Figure 2), scopolamine (Figure 3), and lamotrigine (Figure 4) were added to SH-SY5Y and HT-22 cells for 48 h, in concentrations that varied between 0.01 µM and 20 µM. The results were obtained by MTT and NR assays, as described in the Materials and Methods section. Morphological analysis of both cell lines was also carried out (Figure 5). 

Taken together, our results reveal that overall, mirtazapine, scopolamine and lamotrigine did not decrease SH-SY5Y and HT-22 viability and did not alter the morphology/number of these cells, as evidenced in viability and morphological experiments (Figure 2, Figure 3, Figure 4 and Figure 5). These results demonstrate that these drugs, in these concentrations, were not toxic to the cells. 

### 2.3. Effect of Hydrogen Peroxide and Corticosterone on SH-SY5Y and HT-22 Viability

**Figure 6:**
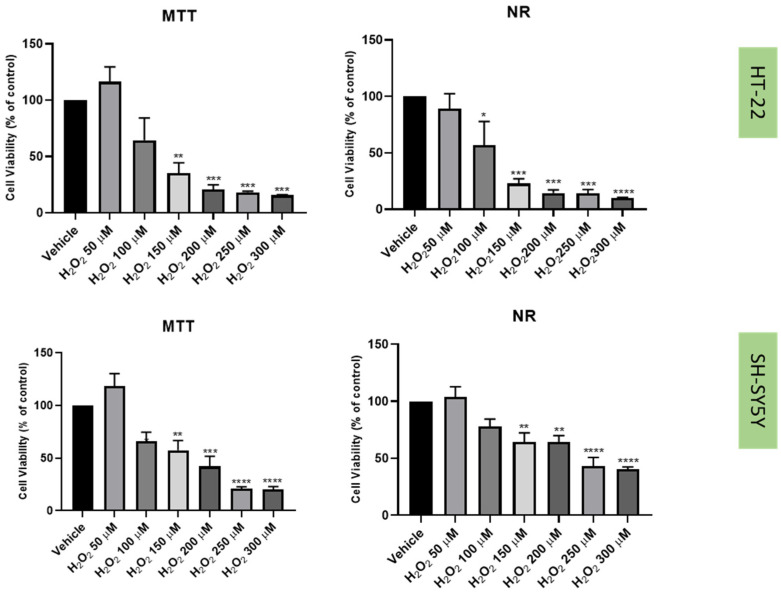
HT-22 and SH-SY5Y viability after incubation of H_2_O_2_ (50 µM–300 µM) for 48 h, obtained by MTT (**left**) and NR (**right**) assays. The results represent the mean ± SEM of 3–6 independent assays (%). Statistical significance at * *p* < 0.05, ** *p* < 0.01, *** *p* < 0.001 and **** *p* < 0.0001 vs. vehicle.

**Figure 7:**
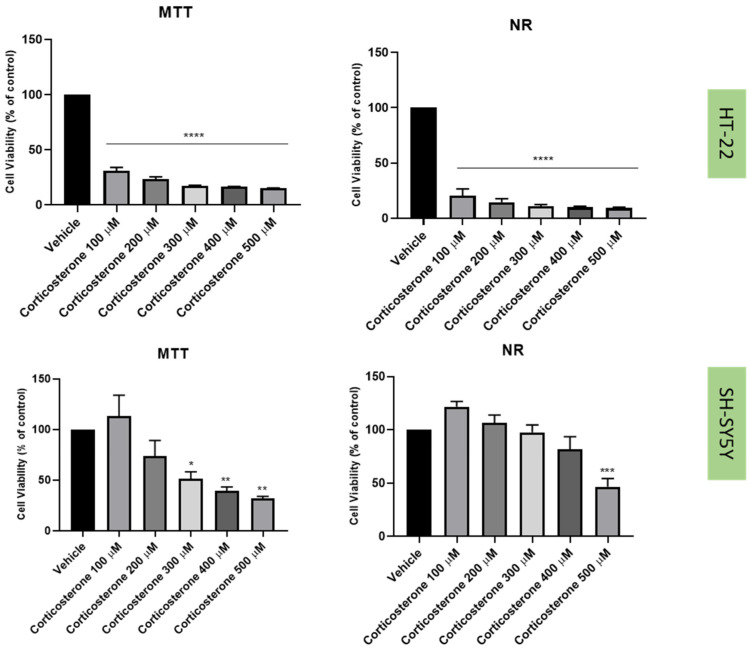
HT-22 and SH-SY5Y viability after incubation of corticosterone (100 µM–500 µM) for 48 h, obtained by MTT (**left**) and NR (**right**) assays. The results represent the mean ± SEM of 3–6 independent assays. Statistical significance at * *p* < 0.05, ** *p* < 0.01, *** *p* < 0.001 and **** *p* < 0.0001 vs. vehicle.

**Figure 8:**
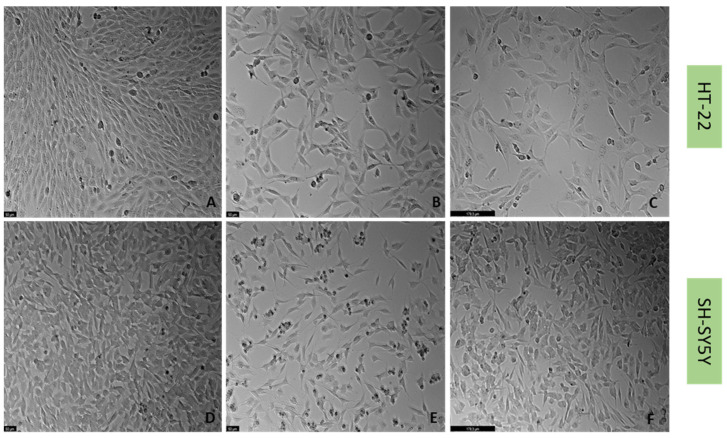
HT-22 and SH-SY5Y morphology after 48 h-incubation of (**A**,**D**) vehicle, (**B**,**E**) H_2_O_2_ 10^5^ µM and 132 µM, respectively, and (**C**,**F**) corticosterone 35 µM and 322 µM, respectively (100× total magnification). Scale bar: 50 µm (**A**,**B**,**D**,**E**) and 179.3 µm (**C**,**F**).

Previously [21], we established a cellular stress model on SH-SY5Y cells with the application of H_2_O_2_ (Figure 6). To understand if this harmful effect was maintained in HT-22 cells, these cells were also treated with crescent concentrations of H_2_O_2_ for 48 h (Figure 6). Additionally, to test the effect of another stressor, both SH-SY5Y and HT-22 cells were incubated with crescent concentrations of corticosterone for 48 h (Figure 7). The results were obtained by MTT and NR assays, as described in the Materials and Methods section. Figure 8 represents morphological observations of both cell lines, after treatment of the cells with the mean of the obtained half-maximal inhibitory concentrations (IC50) values for H_2_O_2_ and corticosterone, by NR and MTT assays.

These results reveal that overall, both H_2_O_2_ and corticosterone decreased HT-22 and SH-SY5Y viability, proportionally to the increase in the concentration, as highlighted in the cell viability assays and morphological assays (Figure 6, Figure 7 and Figure 8). For SH-SY5Y cells, the mean of the obtained IC50 with the application of H_2_O_2_ and corticosterone was 132 µM and 322 µM, respectively. For HT-22, these values were 105 µM and 35 µM, respectively. The concentration-response curves and IC50 determinations are present in the Supplementary Materials (Figures S1 and S2). Together, these data support the stress effect of H_2_O_2_ and corticosterone on both cell lines. 

### 2.4. Effect of Mirtazapine, Scopolamine, and Lamotrigine in Combination with Hydrogen Peroxide and Corticosterone on Cellular Viability

**Figure 9:**
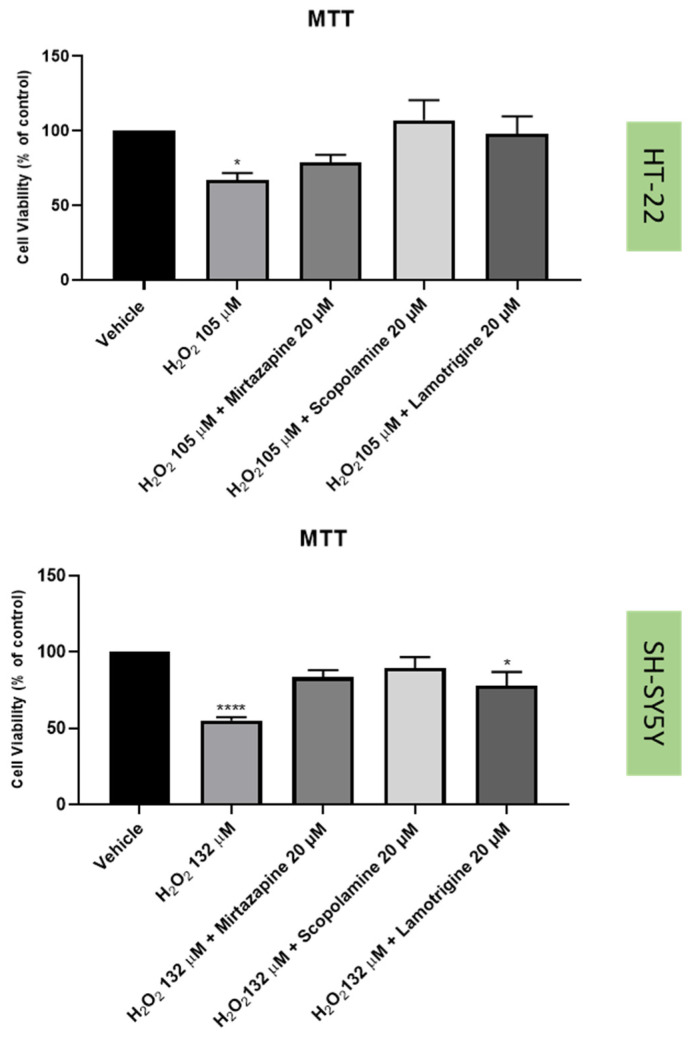
HT-22 and SH-SY5Y viability after incubation of H_2_O_2_ (10^5^ µM and 132 µM) combined with mirtazapine, scopolamine, and lamotrigine (20 µM) for 48 h, obtained by MTT assay. The results represent the mean ± SEM of 3–6 independent assays. Statistical significance at * *p* < 0.05, and **** *p* < 0.0001 vs. vehicle.

**Figure 10:**
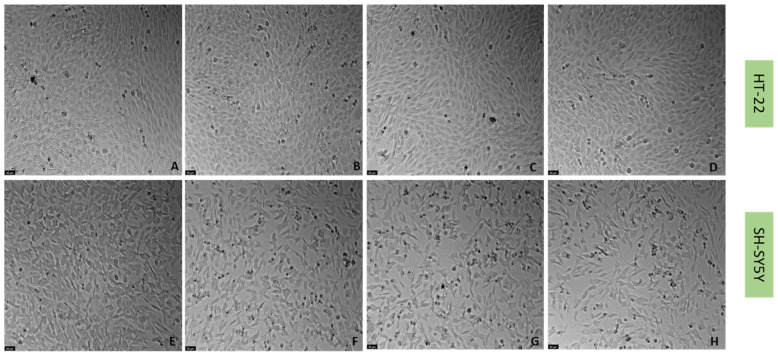
HT-22 and SH-SY5Y morphology after 48 h-incubation of (**A**,**E**) vehicle, (**B**,**F**) H_2_O_2_ 105 µM (for HT-22)/132 µM (for SH-SH5Y) combined with mirtazapine 20 µM, (**C**,**G**) H_2_O_2_ 105 µM (for HT-22)/132 µM (for SH-SH5Y) combined with scopolamine 20 µM, and (**D**,**H**) H_2_O_2_ 105 µM (for HT-22)/132 µM (for SH-SH5Y) combined with lamotrigine 20 µM (100× total magnification). Scale bar: 50 µm.

**Figure 11:**
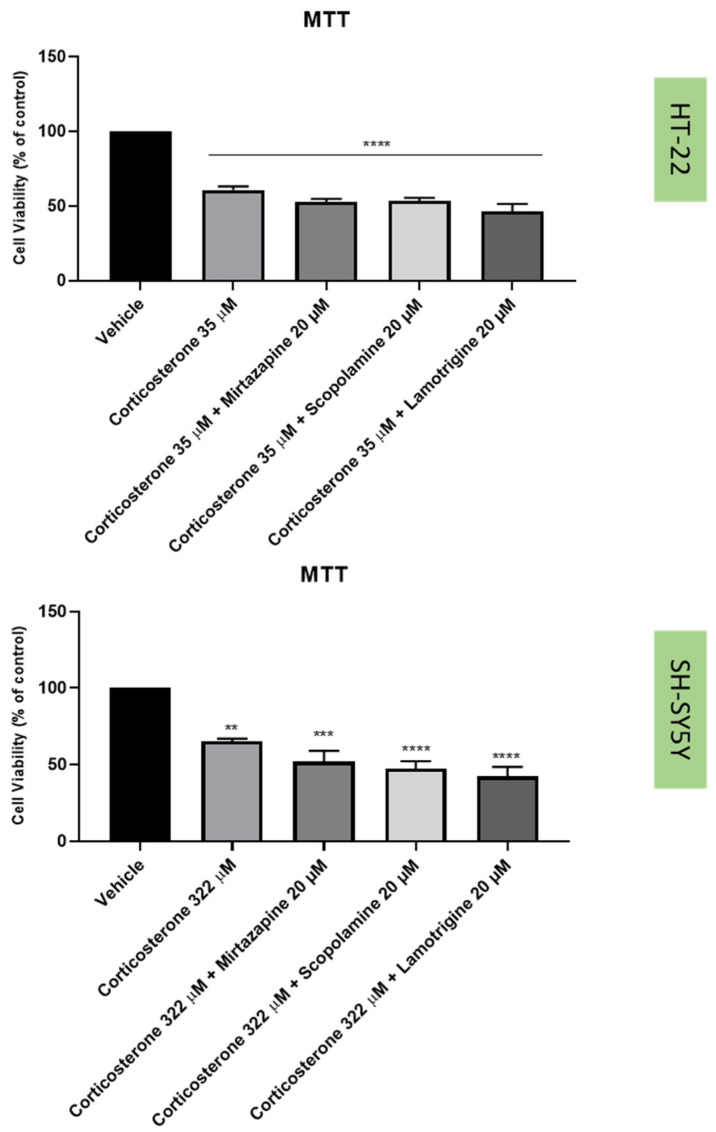
HT-22 and SH-SY5Y viability after incubation of corticosterone (35 µM and 322 µM) combined with mirtazapine, scopolamine, and lamotrigine (20 µM) for 48 h, obtained by MTT assay. The results represent the mean ± SEM of 3–6 independent assays. Statistical significance at ** *p* < 0.01, *** *p* < 0.001, and **** *p* < 0.0001 vs. vehicle.

**Figure 12:**
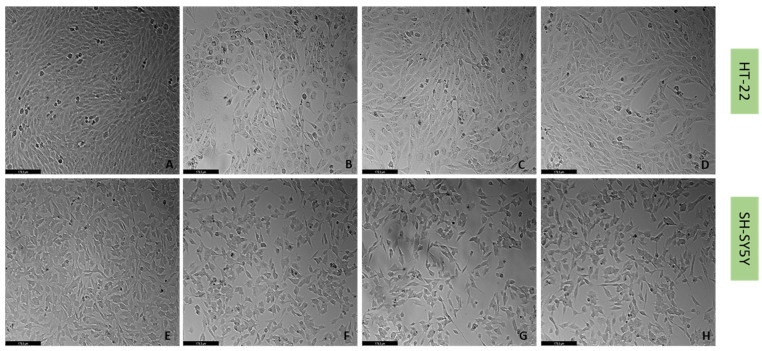
HT-22 and SH-SY5Y morphology after 48 h-incubation of (**A**,**E**) vehicle (**B**,**F**) corticosterone 35 µM (for HT-22)/322 µM (for SH-SH5Y) combined with mirtazapine 20 µM, (**C**,**G**) corticosterone 35 µM (for HT-22)/322 µM combined with scopolamine 20 µM, and (**D**,**H**) corticosterone 35 µM (for HT-22)/322 µM combined with lamotrigine 20 µM (100× total magnification). Scale bar: 179.3 µm.

**Figure 13:**
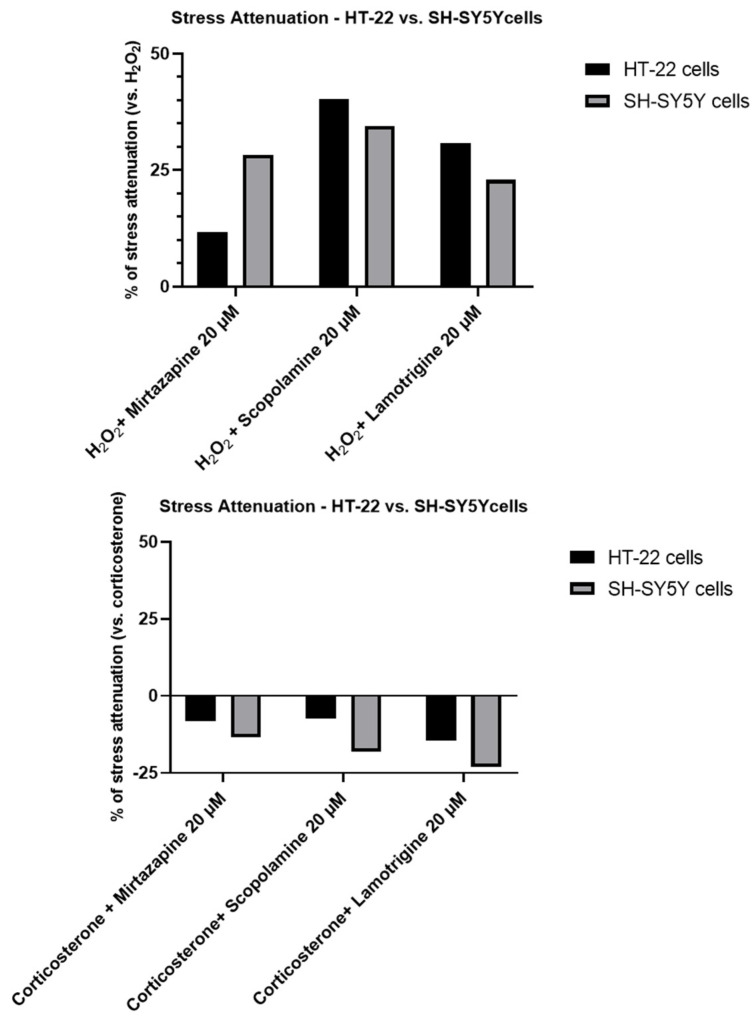
Comparison between the attenuation of H_2_O_2_ and corticosterone-induced stress by mirtazapine, scopolamine, and lamotrigine, in HT-22 and SH-SY5Y cells. The results represent the % of stress attenuation of H_2_O_2_/corticosterone (IC50 values for each cell line) combined with mirtazapine, scopolamine, and lamotrigine (20 µM) in comparison with H_2_O_2_/corticosterone alone (IC50 values for each cell line).

To understand if the three drugs, mirtazapine, scopolamine, and lamotrigine, that interact with the 5-HT3A receptor, could alleviate the harmful effects of H_2_O_2_ (Figure 9 and Figure 10) and corticosterone (Figure 11 and Figure 12) in both SH-SY5Y and HT-22 cells, mirtazapine, scopolamine, and lamotrigine were added to the cells in a concentration of 20 µM, in combination with H_2_O_2_ and corticosterone, for 48 h. After that, morphological evaluation was carried out, and cellular viability was obtained by NR and MTT assays, as described in the Materials and Methods section. H_2_O_2_ was added to SH-SY5Y and HT-22 cells at a concentration of 132 µM and 105 µM, respectively (mean IC50 values). Corticosterone was added to SH-SY5Y and HT-22 cells in a concentration of 322 µM and 35 µM, respectively (mean IC50 values). The comparison between the two cell lines regarding the percentage of stress attenuation is represented in Figure 13. 

Our results evidence that mirtazapine, scopolamine, and lamotrigine attenuated the H_2_O_2_-induced stress in both HT-22 and SH-SY5Y cells (Figure 9, Figure 10 and Figure 13). Indeed, mirtazapine attenuated H_2_O_2_-induced cell stress in approximately 12% (for HT-22) and 28% (for SH-SY5Y), scopolamine attenuated this cell stress in approximately 40% (for HT-22) and 34% (for SH-SY5Y) and, for lamotrigine, the cell stress attenuation was 31% for HT-22 and 23% for SH-SY5Y cells. On the other hand, mirtazapine, scopolamine, and lamotrigine did not attenuate the corticosterone-induced stress in both HT-22 and SH-SY5Y cells (Figure 11, Figure 12 and Figure 13). Both combinations led to viability values (like those obtained with corticosterone alone, or even more decreased). Indeed, mirtazapine increased corticosterone-induced stress by approximately 8% (for HT-22) and 14% (for SH-SY5Y). For scopolamine, this stress was also induced by approximately 7% (for HT-22) and 18% (for SH-SY5Y) and, for lamotrigine, the cell stress also increased in 15% for HT-22 and 23% for SH-SY5Y cells.

### 2.5. Effect of the Drugs and Drug Combinations in Extracellular 5-HT Levels

**Figure 14:**
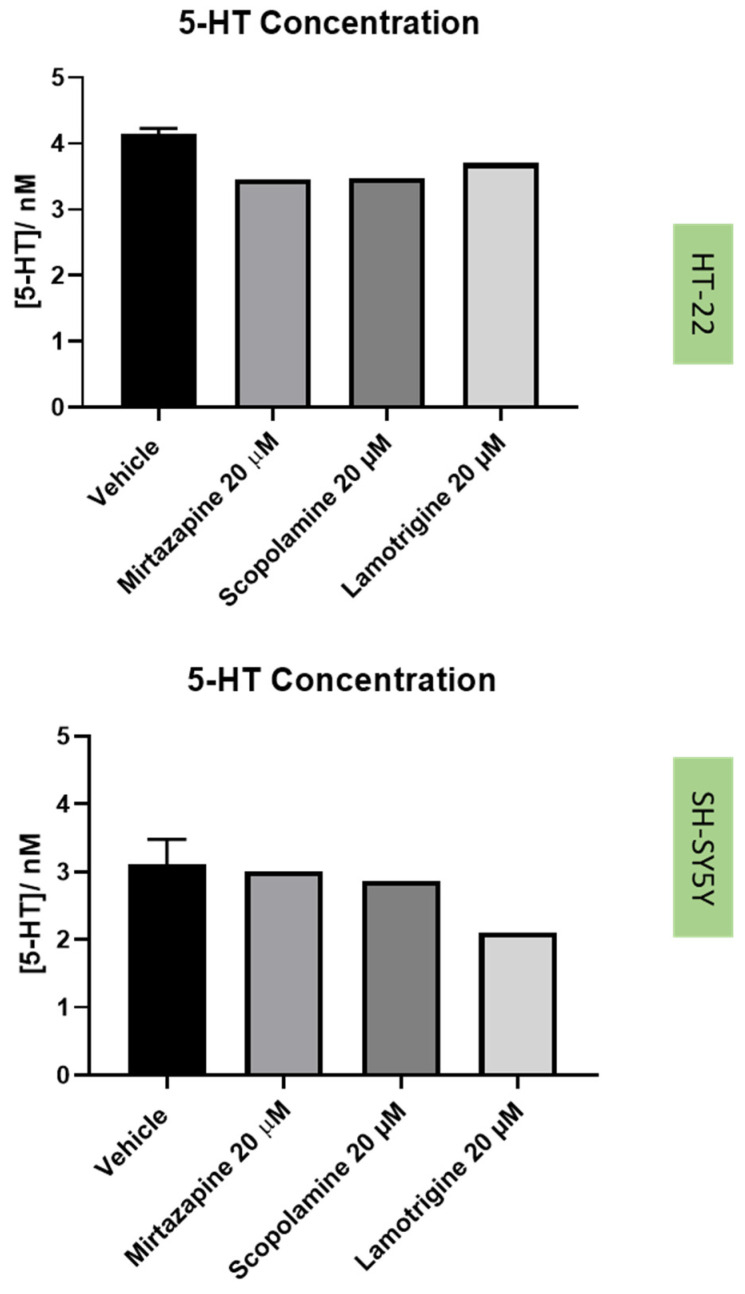
Concentrations of 5-HT (nM) in the extracellular medium of mirtazapine, scopolamine, and lamotrigine-treated HT-22 cells (**above**) and SH-SY5Y cells (**below**), determined by HPLC.

**Figure 15:**
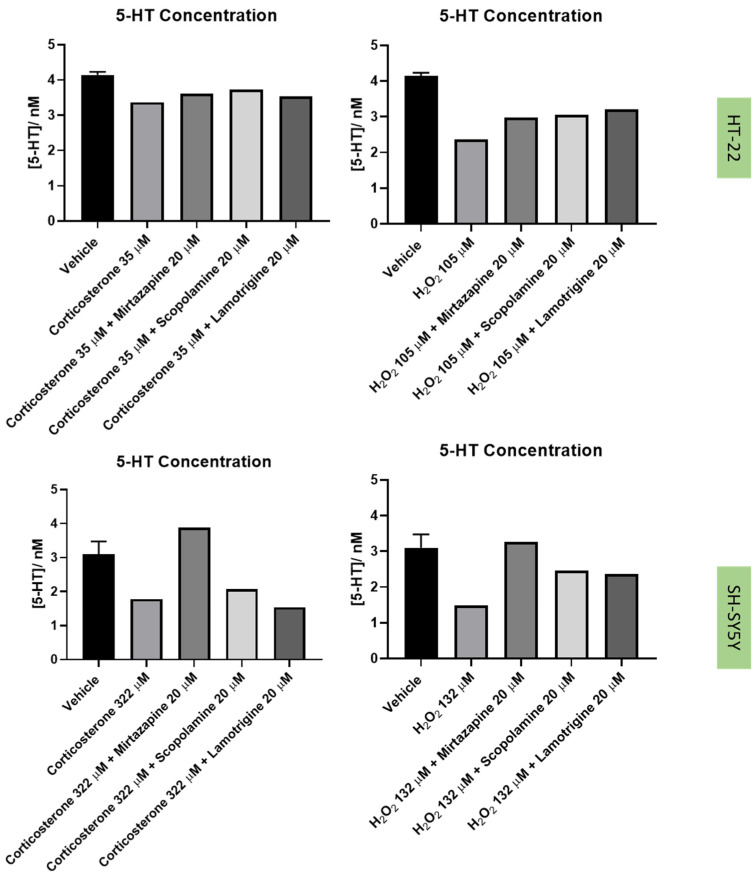
Concentrations of 5-HT (nM) in the extracellular medium of HT-22 cells (**above**) and SH-SY5Y cells (**below**) treated with corticosterone, H_2_O_2_, and their combinations with mirtazapine, scopolamine, and lamotrigine, determined by HPLC.

Next, to understand the effect of the drugs and drug combinations under study in the extracellular 5-HT levels (Figure 14 and Figure 15), 5-HT concentration was determined by HPLC, after 48 h of treatment incubation, as described in the Materials and Methods section. 

## 3. Discussion

Depression represents a worldwide concern. New therapies for this disease and intensive research require urgent implementation [4]. Thus, by using two different neuronal cell lines (SH-SY5Y and HT-22 cells), this work aimed to understand if the receptor 5-HT3 may have an important role in glucocorticoid stress and oxidative stress. To perform these experiments, we used well-known inducers of stress (corticosterone and H_2_O_2_), as well as drugs that are known to interact with 5-HT3 receptor (mirtazapine, lamotrigine, and scopolamine) and that may have the potential to be used in the corticosterone/H_2_O_2_ stress attenuation, as described in the Introduction section. Indeed, our previous work demonstrated that mirtazapine was a good stress reverser for H_2_O_2_, in SH-SY5Y cells [21].

Our results revealed that 5-HT3 receptors were present in both cell lines. To the best of our knowledge, to date, there is no substantial literature that reports the presence of this receptor in these cell lines. Nevertheless, it is known that 5-HT3 is expressed in many brain areas, such as the hippocampus [17]. 

The three drugs that interact with 5-HT3 receptor (mirtazapine, scopolamine, and lamotrigine), in all the tested concentrations, did not lead to cell damage/toxicity, revealing to be good stress reversers in these concentrations. These observations are in concordance with some of the literature, which reports the potential of these drugs to be used in the therapy of depression [26,34,35], as described in the Introduction section. Additionally, HPLC results for these drugs demonstrated that they do not substantially affect 5-HT levels on the extracellular medium, compared to the vehicle, for both cell lines. An exception can be observed with the decreased levels of 5-HT after the application of lamotrigine 20 µM, for SH-SY5Y cells. A hypothesis is that this drug may alter 5-HT levels intracellularly.

Regarding the results obtained with H_2_O_2_, our results revealed that, as expected, this agent led to cellular damage in both SH-SY5Y and HT-22 cells, consistent with previous literature reports [13,36,37]. Additionally, a clear reduction in the 5-HT levels in the extracellular medium was also observed for both cell lines, supporting the hypothesis that H_2_O_2_ disturbs 5-HT-connected pathways in the cells. Indeed, in depression, it is known that suffering individuals present dysfunctions of 5-HT pathways, as well as high levels of oxidative stress [4,38]. Additionally, there are also some evidence that reports that 5-HT protects against oxidative stress in brain [39]. Furthermore, in animals, extracellular levels of 5-HT were observed were increased in active behavioral states and decreased in somnolent periods, demonstrating that extracellular 5-HT levels correlate with depressive behavior. Thus, this data support that high levels of oxidative stress decrease extracellular 5-HT levels, which may correlate with depression [40]. 

The combinations of H_2_O_2_ with mirtazapine, scopolamine, and lamotrigine revealed that these three drugs could increase the levels of extracellular 5-HT, compared with H_2_O_2_ alone, in both cell lines. Additionally, the cellular damage caused by H_2_O_2_ alone was attenuated by the presence of the three drugs, highlighting the possibility that 5-HT3 receptors may be important in the stress reversion of oxidative stress caused by H_2_O_2_. On the other hand, although corticosterone also led to cellular damage in both SH-SY5Y and HT-22 cells (as previously described [41,42]), neither drug alleviated the stress induced by this stress agent, in opposition to the effects observed with H_2_O_2_. These results raise the hypothesis that 5-HT3 receptor may be important in fighting oxidative stress but not glucocorticoid stress. Indeed, corticosterone-induced stress is caused by mechanisms such as the recently described disruption of AMPK/mTOR-mediated autophagy flux [43]. An interesting study revealed that an experimental 5-HT3 receptor antagonist (6z) attenuated brain oxidative damage, demonstrating antidepressant-like activity [19]. However, in another study, an experimental 5-HT3 receptor antagonist (4i) reversed corticosterone induced depressive-like deficits in mice [44]. Additionally, our results also revealed that in SH-SY5Y cells, mirtazapine in combination with corticosterone increased 5-HT levels compared with corticosterone alone, not reflected in the viability results. Thus, more investigation is important in order to clarify the interplay between corticosterone/5-HT3 receptors and discover how this interplay may have a role in depression. 

Analyzing our results, it is also possible to observe that in HT-22 cells, the damage caused by corticosterone is more notable than in SH-SY5Y cells. HT-22 are mice cells and respond better to corticosterone than human cells (SH-SY5Y), because corticosterone is the primary adrenal corticosteroid in mice [45]. Additionally, it is also important to highlight the fact that differences observed between the cell lines can also be explained by the fact that HT-22 cells are hippocampal non-tumoral cells [46], whereas SH-SY5Y are neuroblastoma cells [47]. For example, it was notable that the concentration of 5-HT in the extracellular medium of the cells treated only with the vehicle was different between cell lines, being higher in the non-tumoral HT-22 cells. Indeed, cells use higher concentrations of 5-HT to activate 5-HT-related pathways [48]. As tumoral cells, SH-SY5Y may have higher metabolic activity, having a higher demand of 5-HT [49], which may relate to less availability of 5-HT in the extracellular medium. 

Taken together, our results support the idea that the antagonism of the 5-HT3 receptor by lamotrigine, scopolamine, and mirtazapine may be important in the context of oxidative stress relief in the cells, but not corticosterone-induced stress. This oxidative stress relief was reflected in more cellular viability and more 5-HT concentration in the extracellular medium of the cells, compared with H_2_O_2_ alone. However, future studies to verify the involvement of 5-HT3 receptor in this context are needed. For example, 5-HT3 knockdown/knockout studies in these cells are necessary. Nevertheless, this work evidences the potential role of 5-HT3 receptor in depression. By using cells as the experimental model, this represents a faster and simplified way to study depression, sparing animal use. However, depression is a complex disorder and intense research, including animal studies, must be done in more advanced stages. Nevertheless, in the future, animal study is crucial in order to complement this study.

## 4. Materials and Methods

### 4.1. Materials

From Millipore Sigma (Merck KGaA, Darmstadt, Germany), we purchased Dulbecco’s Modified Eagle’s Medium (DMEM, cat. No. FG0415) and Fetal Bovine Serum (FBS, cat. No. S0615). From Sigma-Aldrich (Merck KGaA, Darmstadt, Germany), we acquired penicillin/streptomycin solution (cat. No. P4333), thiazolyl blue tetrazolium bromide (MTT, cat. No. M5655), neutral red solution (cat. No. N2889), corticosterone (cat. No. 27840), hydrogen peroxide 30% (cat. No. 1.07209), scopolamine hydrobromide (cat. No. PHR1470), laminin (cat. No. L2020), and DAPI (cat. No. D9542). Mirtazapine (cat. No. 19994) and lamotrigine (cat. No. 15428) were acquired from Cayman Chemical Company (Ann Arbor, Michigan, USA). Rabbit polyclonal anti- 5-HT3AR primary antibody (cat. No. ab13897) was obtained from Abcam (Cambridge, United Kingdom), whereas donkey anti-rabbit 488 secondary antibody was purchased from Molecular Probes (Waltham, OR, USA). Donor horse serum (cat. No. S0900) was obtained from Biowest (Nuaillé, France), poly-D-lysine (cat. No. A3890401) was purchased from Gibco (Thermo Fisher Scientifics, Waltham, MA, USA), prolong gold antifade reagent (cat. No. P10144) was purchased from Invitrogen (Eugene, MA, USA), and for the HPLC procedure, waters alliance 2695 pump and 3030 reagent kit^®^ were obtained from Waters Corporation (Milford, MA, USA), rheodyne loop injector was purchased from Chromsystems GmbH (Munich, Germany), and from Antec Scientific (Zoeterwoude, The Netherlands), we acquired the Decade electrochemical detector (ECD).

### 4.2. Cell Treatments

Mirtazapine and H_2_O_2_ were prepared and added to the cells as previously described [21]. Corticosterone and lamotrigine were dissolved in DMSO (or methanol, for HPLC analysis purposes), 0.1% in the cell culture medium. Scopolamine was prepared in sterilized water (0.1% in culture medium). Mirtazapine, lamotrigine, and scopolamine were added to the cells in concentrations ranging between 10 nM and 20 µM, depending on the experiment. Corticosterone and H_2_O_2_ were added to the cells in concentrations ranging 100 µM–500 µM and 50 µM–300 µM, respectively. For mirtazapine, lamotrigine, scopolamine, and corticosterone alone, the vehicle was composed of 0.1% DMSO in the cell culture medium, whereas for scopolamine and H_2_O_2_ alone, the vehicle was composed of 0.1% sterilized water in cell culture medium. For mirtazapine and lamotrigine combined with H_2_O_2_, vehicles were composed of 0.1% sterilized water/0.1% DMSO or methanol. For scopolamine combined with H_2_O_2_, the vehicle was 0.2% sterilized water. For corticosterone combined with mirtazapine and lamotrigine, the vehicle was 0.2% DMSO or methanol and, finally a vehicle of 0.1% DMSO or methanol/0.1% sterilized water was used for corticosterone combined with scopolamine. All the drugs were added to the cells for a period of 48 h.

### 4.3. Cell Culture

The SH-SY5Y cell line (American Type Culture Collection, VA, USA), and HT-22 cells (provided by Professor Ana Cristina Rego’s group, University of Coimbra, Portugal) were incubated at 37 °C (95% air, 5% CO_2_) in DMEM (10% FBS, 1% penicillin (1000 U/mL)/streptomycin (10 mg/mL)). Trypsinization (0.25% trypsin-EDTA) and centrifugation (800 rpm for HT-22 and 1100 rpm for SH-SY5Y, 5 min; Hettich, Tuttlingen, Germany) were carried out before each cellular assay. Cells were seeded at a density of 1.0 × 10^5^ cells/mL (SH-SY5Y cells) and 1.5 × 10^4^ cells/mL (HT-22 cells) in 96-well plates (200 μL/well), or 1.3 × 104 cells/mL in 24-well plates (500 µL/well).

### 4.4. Immunofluorescence 

HT-22 and SH-SY5Y cells were grown on 24-well plates, covered with 13-mm coverslips, and coated with poly-D-lysine (20 μg/mL in PBS-1x) and laminin (5 μg/mL in PBS-1x). After that, the cells were washed with PBS-1x and plated at a seeding density of 6500 cells/well. Then, cells were fixed with 4% paraformaldehyde at room temperature for 10 min and washed 3 × 5 min with 0.1% Triton X-100 in PBS-1x. Next, cells were blocked with donor horse serum 5% in PBST (PBS-Tween 20 0.1%), for 1 h, at room temperature. After that, the primary antibody rabbit polyclonal anti-5-HT3AR) was diluted in donor horse serum 5% in PBST and added to the cells in a ratio of 1:500, following an overnight period of incubation. Then, cells were washed with 0.1% Triton X-100 in PBS-1x (3 × 5 min) and incubated for 1 h at room temperature with the donkey anti-rabbit 488 secondary antibody (1:1000, diluted in donor horse serum 5% in PBST). Finally, cells were washed with 0.1% Triton X-100 in PBS-1x (3 × 5 min), incubated with DAPI (1:1000 in PBS-1x) for 10 min, and washed twice with PBS-1x. For mounting, ProLong^TM^ gold antifade mountant was used on each slide. Images were acquired on ApoTome Slider (Zeiss^®^) fluorescence microscope coupled to the AxioVision Rel. 4.8. software (Zeiss^®^).

### 4.5. Cell Viability Assays

To evaluate the cell viability after the different treatments (48 h), MTT assay and neutral red (NR) assay were carried out as previously described [21]. Briefly, for the MTT assay, MTT (0.5 mg/mL in PBS) was added to the cells (100 µL) and the plate was incubated for 3 h (37 °C). Then, this reagent was removed and DMSO was added to the cells (100 µL). The 570 nm absorbance values were obtained from the automated microplate reader (Tecan Infinite M200, Zurich, Switzerland). For the NR assay, NR medium (1:100 in DMEM) was added to each well (100 µL) and the plate was incubated for 3 h (37 °C). After that, the cells were washed in PBS and NR destain solution (50% of 96% ethanol, 49% deionized water, and 1% glacial acetic acid) was added to the cells (150 µL). The 540 nm absorbance values were obtained in the automated microplate reader. 

### 4.6. Cell Morphology Assessment 

The cellular morphology of SH-SY5Y and HT-22 cell lines was assessed by using Leica DMI6000 B Automated Microscope (Wetzlar, Germany) after all the treatment conditions before the cell viability assays. 

### 4.7. HPLC Analysis

HPLC analysis was carried out as previously described [50]. Briefly, after being filtrated and centrifugated, the analysis of 5-HT content in the samples was performed using the 3030 Reagent kit for HPLC analysis of 5-HT in the urine. The calibration curve was generated with concentrations between 1–1000 nM of 5-HT. This curve was used to calculate the concentration of 5-HT in each sample, as previously described [50]. All the samples were collected after 48 h of the cell treatments. 

### 4.8. Statistical and Data Analysis 

Statistical and data analysis was performed using the software GraphPad Prism 8 (San Diego, CA, USA). Statistical comparisons between vehicle and treatment groups were performed with one-way ANOVA and Dunnett’s multiple comparisons test. Statistical significance was obtained when *p* < 0.05. All the cell viability studies results represent the mean ± SEM of 3–6 independent assays.

## Data Availability

Not applicable.

## Supplementary Materials

The following supporting information can be downloaded at: www.mdpi.com/article/10.3390/life12101645/s1, Figure S1: Concentration-response curves of H_2_O_2_ and corticosterone for SH-SY5Y cells, obtained by MTT (left) and NR (right) assays, for 48 h. The results represent the mean ± SEM of 3–6 independent experiments; Figure S2: Concentration-response curves of H_2_O_2_ and corticosterone for HT-22 cells, obtained by MTT (left) and NR (right) assays, for 48 h. The results represent the mean ± SEM of 3–6 independent experiments.
