# Supplementary material for: Serotonin Type 3 Receptor Is Potentially Involved in Cellular Stress Induced by Hydrogen Peroxide
Source: Life (Basel). 2022 Oct 19;12(10):1645. doi: 10.3390/life12101645 (PMC9605598; doi:10.3390/life12101645)
Supplement: Supplementary file 1 [file life-12-01645-s001.zip › life-1965119-supplementary.pdf]

Article

Serotonin Type 3 Receptor Is Potentially Involved in Cellular Stress Induced by Hydrogen Peroxide

Ana Salomé Correia [1,2,3], Isabel Silva [4], José Carlos Oliveira [4,5], Henrique Reguengo [4,5] and Nuno Vale [1,3,6] *

[1] OncoPharma Research Group, Center for Health Technology and Services Research (CINTESIS), Rua Doutor Plácido da Costa, 4200-450 Porto, Portugal
[2] Institute of Biomedical Sciences Abel Salazar (ICBAS), University of Porto, Rua de Jorge Viterbo Ferreira 228, 4050-313 Porto, Portugal
[3] CINTESIS@RISE, Faculty of Medicine, University of Porto, Alameda Professor Hernâni Monteiro, 4200-319 Porto, Portugal
[4] Clinical Chemistry, Department of Laboratory Pathology, Hospital Center of the University of Porto (CHUP), Largo Prof. Abel Salazar, 4099-313 Porto, Portugal
[5] Unit for Multidisciplinary Research in Biomedicine (UMIB), University of Porto, Rua de Jorge Viterbo Ferreira, 228, 4050-313 Porto, Portugal
[6] Department of Community Medicine, Health Information and Decision (MEDCIDS), Faculty of Medicine, University of Porto, Rua Doutor Plácido da Costa, 4200-450 Porto, Portugal
*Correspondence: nunovale@med.up.pt

Supplementary Materials

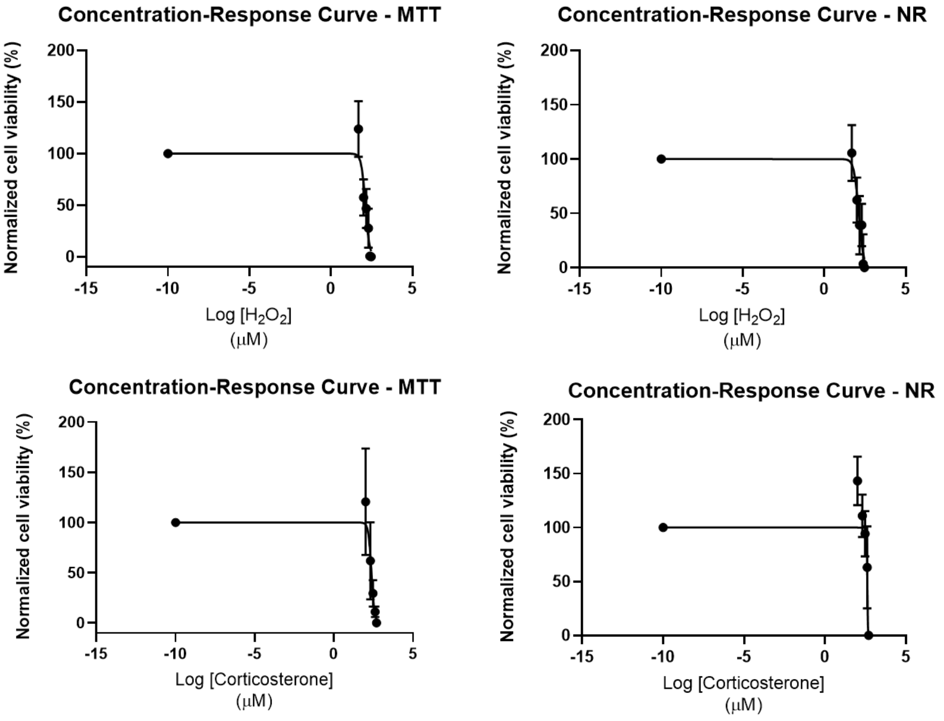

Figure S1. Concentration-response curves of H_2O_2 and corticosterone for SH-SY5Y cells, obtained by MTT (left) and NR (right) assays, for 48h. The results represent the mean ± SEM of 3-6 independent experiments.

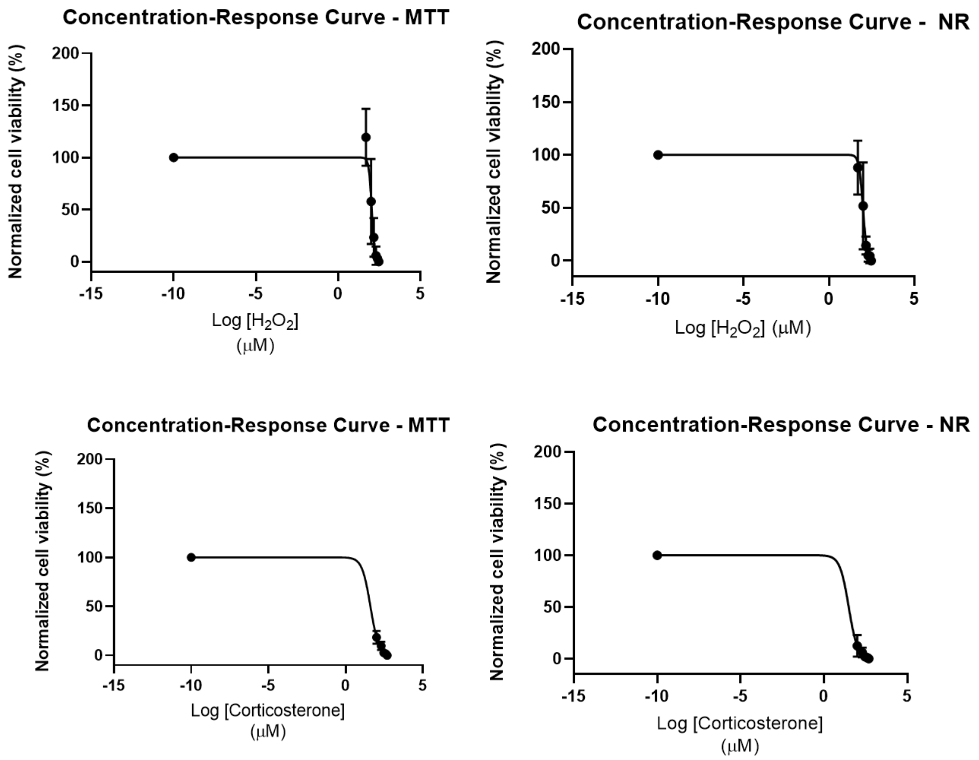

Figure S2. Concentration-response curves of H_2O_2 and corticosterone for HT-22 cells, obtained by MTT (left) and NR (right) assays, for 48h. The results represent the mean ± SEM of 3-6 independent experiments.
